# Recruitment of Adolescents to Virtual Clinical Trials: Recruitment Results From the Health4Me Randomized Controlled Trial

**DOI:** 10.2196/62919

**Published:** 2024-12-16

**Authors:** Rebecca Raeside, Allyson R Todd, Sarah Barakat, Sean Rom, Stephanie Boulet, Sarah Maguire, Kathryn Williams, Seema Mihrshahi, Maree L Hackett, Julie Redfern, Stephanie R Partridge, The Health4Me Team, Katharine Steinbeck

**Affiliations:** 1 Susan Wakil School of Nursing and Midwifery, Faculty of Medicine and Health, The University of Sydney, Western Avenue, Camperdown, 2050, Australia, 61 0468684450; 2 InsideOut Institute for Eating Disorders, Faculty of Medicine and Health, The University of Sydney, Sydney, Australia; 3 Department of Endocrinology, Nepean Hospital, Nepean Blue Mountains Local Health District, Kingswood, Australia; 4 Department of Health Sciences, Faculty of Medicine, Health and Human Sciences, Macquarie University, Sydney, Australia; 5 The George Institute for Global Health, University of New South Wales, Sydney, Australia; 6 Institute for Evidence-Based Healthcare, Bond University, Robina, Australia; 7 Charles Perkins Centre, The University of Sydney, Camperdown, Australia; 8 see Acknowledgments

**Keywords:** adolescents, clinical trial, recruitment, digital health, prevention, adolescent health, health behavior change, health promotion, social media

## Abstract

**Background:**

Preventive interventions are needed to provide targeted health support to adolescents to improve health behaviors. Engaging adolescents in preventive interventions remains a challenge, highlighting the need for innovative recruitment strategies. Given adolescents’ lives are intertwined with digital technologies, attention should be focused on these avenues for recruitment. The evolving nature of clinical trials, including the emergence of virtual clinical trials, requires new recruitment approaches, which must be evaluated.

**Objective:**

This study aimed to examine the effectiveness and cost of various digital recruitment strategies for recruiting adolescents to a virtual clinical trial, evaluate the progression of participants from screening to enrollment, and explore factors associated with nonparticipation. This was conducted using data from the Health4Me Study, a preventive digital health intervention to improve physical activity and nutrition behaviors among adolescents aged 12 to 18 years.

**Methods:**

Participants were recruited into the Health4Me Study via social media advertisements on various contemporary platforms, emails to schools, emails to contacts within known networks, and emails to relevant youth organizations. Data were collected from social media advertisements, screening, and recruitment logs. Data analysis included summary and descriptive statistics, as well as chi-square tests to explore factors associated with nonparticipation.

**Results:**

From 2369 expressions of interest, 390 (16.4%) participants were enrolled. A total of 19 advertisements were placed on social media, and 385 promotional emails were sent to schools, contacts within known networks, and relevant youth organizations. Social media advertisements reached 408,077 unique accounts. Advertisements mostly reached those living in populous states in Australia (306,489/408,077, 75.11% of unique accounts) and those identifying as female (177,698/408,077, 43.55% of unique accounts). A total of 24.97% (101,907/408,077) of advertisements were delivered to accounts with uncategorized genders. The total cost per participant enrolled was Aus $3.89 (approximately US $2.58). Most participants (1980/2305, 85.90%) found out about this study through Instagram. Differences in screening characteristics between eligible participants who did and did not enroll were found to be statistically significant for gender (*P*=.02), with fewer males and more individuals reporting their gender as “other” enrolling than expected by chance alone. The recruitment method also differed (*P*<.001), with fewer participants enrolling through Instagram and more enrolling through other methods (eg, known networks or word of mouth) than expected by chance alone.

**Conclusions:**

This study found that virtual clinical trial recruitment was found to be low-cost, with the potential to increase trial participation. Social media was the most effective recruitment method, reaching all states and territories, including hard-to-reach populations. Future action is needed to explore recruitment methods that are more effective for males and to build trust among adolescents regarding clinical trial recruitment via social media.

## Introduction

Adolescence is regarded as the second window of opportunity—a critical period to intervene and provide targeted support to improve health outcomes that have a profound impact on health and well-being throughout the life course [1]. Failure to invest in primary prevention among today’s adolescents will increase the burden of chronic diseases and the existing sizable total health expenditure of Aus $24 billion (approximately US $15.9 billion) on potentially avoidable risk factors [2]. It is important that high-quality public health interventions that focus on primary prevention of chronic diseases are tested with adolescents through clinical trials. However, challenges exist with engaging adolescents in preventive interventions, including their health system disengagement making them hard to access for delivering such interventions [3], a prevention lens not being appealing to adolescents [4], and their evolving need for autonomy in providing informed consent [5]. Innovative methods to engage adolescents within preventive interventions are needed that can overcome identified barriers.

Adolescents’ lives are increasingly intertwined with digital technologies such as mobile phones and the internet [6]. With that comes the opportunity to harness developments in digital technologies for innovative preventive interventions [7]. The use of digital methods for recruitment to clinical trials is increasing in popularity and they are particularly beneficial for recruitment to online clinical trials. Previous research has focused on comparing social media or other digital strategies to traditional in-person recruitment [8-13], and the use of digital tools for recruitment and retention of clinical trial participants [14,15]. While research shows that digital recruitment strategies are effective compared to traditional in-person recruitment, limited research is available to understand the efficiency of digital recruitment strategies alone and their impact on clinical trial participants and investigators (eg, helping investigators identify eligible trial participants) [14]. A previous review identified that Facebook (Meta) is effective for recruiting adolescent participants [16], and a cross-sectional study revealed that the use of Instagram (Instagram from Meta) and Snapchat (Snap Inc) may also be useful and cost-effective to recruit young people to surveys [17], but limited evidence is available for the use of these contemporary platforms for adolescent recruitment to clinical trials. As more social media platforms become available and others diminish in popularity, it is crucial that research is undertaken to understand their effectiveness for recruiting adolescents to research.

Additional complexities occur when there are no physical recruitment sites, otherwise known as remote [18], decentralized [19], or virtual [20] clinical trials, from hereon in called virtual clinical trials. Virtual clinical trials can leverage digital technologies for participant recruitment and retention, enabling online consent for participants, on-time data collection, and delivery of the intervention that is convenient for participants, as they do not have to travel to a physical site [21]. Virtual clinical trials among adolescent participants have the potential to overcome some of the previously identified barriers, including reaching those who are disengaged with the health system and reaching adolescents directly [22,23], allowing them autonomy in making decisions about their health [24], including providing informed consent (depending on ethics approvals). However, there is limited research to understand digital recruitment strategies for clinical trials among adolescents. Furthermore, it is also important to understand factors that may cause eligible participants not to engage in digital preventive interventions. Reporting will enable future research to tailor recruitment toward the most effective digital strategies and address factors that cause disengagement. Therefore, this study aimed (1) to examine the effectiveness and cost of various digital recruitment strategies for recruiting adolescents to a virtual clinical trial, (2) to evaluate the progression of participants from screening to enrollment, and (3) explore factors associated with nonparticipation.

## Methods

### Study Design

The Health4Me study was used as the context for this research. The full protocol is published elsewhere [25]. In brief, the Health4Me Study is a virtual clinical trial, based in Australia, of a community-based, 6-month text message intervention. The intervention aims to improve physical activity and nutrition behaviors among adolescents aged 12 to 18 years.

### Ethical Considerations

Primary ethics approval was received from the University of Sydney Human Research Ethics Committee (2022/402), and the trial is registered at the Australia New Zealand Clinical Trials Registry (ANZCTR; ACTRN12622000949785; date registered: July 5, 2022).

### Participants and Eligibility Criteria

Participants were eligible to take part in the Health4Me Study if they were (1) aged 12 to 18 years, (2) owned a mobile phone capable of sending and receiving text messages, (3) had an Australian mobile phone number, (4) had sufficient English proficiency to read text messages pitched at a 7th grade reading level, and (5) provided electronic consent (or from their parents or guardians if they were aged <14 years). Participants were excluded from this study if they (1) had a diagnosis of type 1 or type 2 diabetes mellitus, (2) had a previous or current diagnosis of an eating disorder or were at high risk for an eating disorder as assessed in screening, (3) weighed <25th centile for their age, (4) had recent rapid weight loss, (5) had a medical condition that would preclude providing informed consent or ability to comply with this study’s protocol, (6) were enrolled in an alternative randomized lifestyle management program, (7) were pregnant or planning to become pregnant during the 6-month intervention, and (8) were unable to read English at a 7th grade reading level. The eligibility criteria for the Health4Me Study have been published elsewhere [25].

Given the Health4Me Study was conducted virtually, several steps were embedded to ensure participants could safely enroll into this study. To complete screening procedures, the research team partnered with the InsideOut Institute for Eating Disorders, a team of researchers and clinician experts in eating disorders based at the University of Sydney. Potential participants first expressed interest to take part in this study by filling out the Expression of Interest (EOI) form on REDCap (Research Electronic Data Capture; Vanderbilt University), which included contact details and screening against the eligibility criteria, as well as screening for eating disorder risk using two validated questionnaires—InsideOut Institute Screener (IOI-S) and Eating Disorder Examination Questionnaire (EDE-Q) [26,27]. Study specific cut points were set for the IOI-S (≥16) and EDE-Q (>3 and any of behavioral questions 15‐18 endorsed ≥1). Potential participants first completed the IOI-S, if they scored below the cut point and met all inclusion criteria, they were sent the e-consent form. However, if participants scored above the cut point on the IOI-S, they were directed to complete an EDE-Q. If potential participants scored under the cut point on the EDE-Q, they were deemed eligible and sent the e-consent form. If a potential participant was detected to be above the cut point on the EDE-Q, they were referred to the InsideOut Institute for Eating Disorders for an assessment to determine suitability to participate by eating disorder expert clinicians (clinical psychologist or registered clinical psychology students with expertise in eating disorders) via phone call. If they received clearance from the eating disorder expert clinicians, they were sent the e-consent form and deemed eligible to enroll in this study. If they did not receive clearance, they were sent an email with various resources for eating disorder support. If a potential participant did not meet other inclusion criteria, they were sent an email explaining why they were ineligible. All participants provided informed e-consent (and from their parents or guardians if they were aged <14 years) before baseline measures were collected [25]. Participants were randomized once all baseline measures were complete.

### Recruitment

The protocol was to enroll 390 participants—195 per arm—based on detecting a mean difference in moderate to vigorous physical activity minutes per day of 14.8 (control: 42.55 and intervention: 57.36) with an SD of 21.45 for control and 37.79 for intervention or a 13.37% difference in the proportion of appropriate vegetable consumption (control: 4.85% and intervention: 18.22%) with 90% power and accounting for 30% dropout. The Bonferroni adjusted significance level of 0.025 was used to account for two primary outcomes. The participant information statement detailed that participants would receive an Aus $30 (approximately US $19.90) gift voucher at the completion of all baseline assessments as a reimbursement for their time. A recent review has suggested that financial incentives can be provided to children appropriately, and few studies suggest incentives are inherently harmful [28]. Recruitment methods are detailed below.

### Recruitment Methods

#### Overview

Recruitment ran from February 2023 to February 2024 using a range of methods including social media advertising on Facebook, Instagram, TikTok, and Twitter/X, emails to schools, emails to contacts within known networks, and emails to relevant youth organizations. A dedicated study website was also created to establish legitimacy.

#### Social Media Advertisements

Initially, study-dedicated Facebook and Instagram pages were established with this study’s logo, study contact information and detailed the purpose of this study. Posts were made on Instagram to establish this study as an authentic and active social media account. All content and this study logo were co-designed with adolescents [29]. All advertisements were created using ethics approved text and images on Meta Ads Manager, which simultaneously promoted advertisements on Facebook and Instagram or on TikTok for Business, which promoted advertisements on TikTok. Due to restrictions in advertising to people aged younger than 18 years [30], all advertisements were targeted only for people aged 13 to 18 years in Australia. Examples of the social media advertisements (images and text) are available in Multimedia Appendix 1. Advertisements on Meta were run for a maximum of 2 weeks, with a maximum budget of Aus $20 (approximately US $13.30) per day. The single advertisement on TikTok was run for 4 days, with a lifetime budget of Aus $50 (approximately US $33.20). A single post was made on Twitter/X by a member of the research team. All advertisements linked directly to this study EOI form, hosted on REDCap.

#### Emails

Emails were sent to schools, known networks and contacts of the research team and, relevant youth organizations, for example, headspace. Emails contained a link to this study’s website and this study’s REDCap page.

#### Study Website

Previous formative work by the research team revealed that adolescents desire online health information that is credible and reliable [31]. A study website was created to establish this study as legitimate and to build trust among adolescents. This study’s website contained this study’s logo, study contact information, detailed the purpose of this study, how to become involved (including a direct link to this study’s REDCap page), photographs and names of the key researchers and names of the wider research team. Potential participants could also access the full participant information sheet through this study’s website.

### Data Sources

#### Social Media Advertisements

Data were available and collected from Meta Ads Manager. For each advertisement, data were collected on the number of days the advertisement ran, advertisement strategy used, reach, impressions, link clicks, cost per result, and total amount spent (Aus $). Deidentified advertisement audience demographic data included location, age, and gender. User’s location was based on their state or territory (New South Wales [NSW], Victoria, South Australia, Queensland, Western Australia, Northern Territory, Tasmania, and Australian Capital Territory). Age and gender data were based on what social media users disclose on their user profiles and were summarized according to Meta Ads Manager categories (age: 13‐17 or 18‐24 years; gender: male, female, or uncategorized). Data were available and collected from TikTok for Business. For each advertisement, data were collected on the number of days the advertisement ran, reach, impressions, link clicks, cost per result, and total amount spent (Aus $). Post analytics were available and collected from Twitter/X. For the single post, data were collected on likes, reposts, impressions, and link clicks.

#### Recruitment Log

A log was kept of all dates on which emails were sent to schools, known networks and contacts, and youth organizations. Data were also collected on the number of people who visited this study’s REDCap page each day to express interest and the number of enrollments. Detailed notes were kept on the log by the research team.

#### Screening Logs

A detailed log was kept of all participant inquiries. The secure online REDCap [32] database collected data, including age (12-14 years or 15-18 years), gender (male, female, other, or prefer not to say), high school attendance (yes or no), height and weight (for BMI calculations, categorized as underweight, healthy weight, above a healthy weight, or well above a healthy weight) [33,34], and recruitment method. The responses for recruitment method included (1) Facebook, (2) Instagram, (3) Twitter/X, (4) TikTok, (5) other social media platform, (6) headspace, (7) general practitioner or doctor, and (8) other. The screening log also contained details on eligibility and reasons for exclusion. A further screening log was also collected from the InsideOut Institute for Eating Disorders, which kept a detailed log of potential participants requiring screening for eating disorder risk. The secure online REDCap database allowed both the psychologists and research team to make comments. Potential participants were contacted a maximum of two times by the eating disorder expert clinicians. If contact was not established after two attempts, they were marked as ineligible and sent resources via email.

### Data Analysis

Summary statistics regarding social media data are presented. Total costs (Aus $) are reported for social media advertisements, with the average cost calculated per participant eligible and per participant enrolled. Descriptive statistics for continuous measures, including counts and percentages for recruitment method, were used to summarize the breakdown of potential participants who inquired and participants screened by the InsideOut Institute for Eating Disorders.

To explore factors associated with nonparticipation, differences in screening characteristics between eligible participants who did and did not enroll in this study were compared using chi-square tests. The significance level was set at 5%. Characteristics included age, gender, BMI, high school attendance, and recruitment method. Adjusted standardized residuals (ASRs) were used to measure the strength of the difference between observed and expected values. Data were analyzed using IBM SPSS (version 29.0; IBM Corp).

## Results

### Effectiveness and Cost of Recruitment Strategies

A total of 2369 entries were made to the EOI form. Of those, 2305 respondents completed the question asking how they heard about this study. Most (1980/2305, 85.90%) found out about this study through Instagram, followed by other (182/2305, 7.9%) and then Facebook (112/2305, 4.9%). The full sample size of 390 adolescents was reached in 12 months.

For the Health4Me Study, there were 17 advertisements run on Meta Ads Manager over 12 months. The length at which the advertisements were running for ranged from 2‐18 days, with advertisements running for a total of 146 days. Overall, advertisements reached a total of 408,077 unique Meta accounts and were viewed >2.3 million times. A cost-per-link-click strategy was employed in 16/17 advertisements, and one employed a cost-per-post-engagement strategy. Across 17 advertisements, 7211 link clicks were made. Advertisements mostly reached people in NSW, Victoria, and Queensland, accounting for 75.11% (306,489/408,077) of the audience. With regard to age, 96.89% (395,403/408,077) of the advertisement audience was 13‐17 years old. For gender, advertisements mostly reached females (43.55%, 177,698/408,077); however, a quarter (24.97%, 101,907/408,077) of the advertisements were delivered to accounts with uncategorized genders. One advertisement was run through TikTok for Business, which ran for 4 days, reached 8386 unique accounts, and was viewed 14,832 times, with 144 link clicks made. No further data were available. One advertisement was placed on Twitter/X, which was viewed a total of 1041 times and reposted 11 times, with 15 link clicks made.

The overall cost of social media advertisements run through Meta was Aus $1478.63 (approximately US $965.69). Cost-per-link-click ranged from Aus $0.03 to Aus $0.87 (approximately US $0.02 to US $0.54). The overall cost of the advertisement through TikTok was Aus $39.97 (approximately US $26.10), with cost-per-link-click at Aus $0.28 (approximately US $0.18). In total, Aus $1518.60 (approximately US $991.80) was spent on social media advertisements. Cost per eligible participant was Aus $1.64 (approximately US $1.09), and cost per participant enrolled was Aus $3.89 (approximately US $2.58). A full breakdown of all social media data is available in Tables1  2.

Emails requesting inclusion in school communications to students were sent to 367 high schools across NSW. One school announced this study at their school assembly. One email was sent to our mailing list of young people who have expressed interest in future research. Emails were sent to 17 other known networks, contacts, and youth organizations. Of those, the research team was made aware that one shared in their general practitioner newsletter, and one shared within their local health district. All sharing through schools, known networks, contacts, and youth organizations was at no cost to the research team.

**Table 1.** Breakdown of Meta advertisements reach by state or territory, age, and gender.

	Reach, n (%)
**State or territory**	
New South Wales	123,543 (30.27)
Victoria	95,954 (23.51)
Queensland	86,992 (21.32)
Western Australia	44,808 (10.98)
South Australia	31,814 (7.8)
Northern Territory	5633 (1.38)
Tasmania	9858 (2.42)
Australian Capital Territory	5377 (1.32)
Unknown	4098 (1)
**Age (years)**	
13‐17	395,403 (96.89)
18‐24	12,674 (3.11)
**Gender**	
Female	177,698 (43.55)
Male	128,472 (31.48)
Uncategorized	101,907 (24.97)
Total	408,077 (100)

**Table 2.** Breakdown of social media advertisements for the Health4Me study through Meta, TikTok, and Twitter/X.

Social media platform and advertisement start date		End date	Days advertisements live (n)	Advertisement strategy^a^	Reach^b^	Impressions^c^	Total cost (Aus $^d^)	Daily budget (Aus $)	Link clicks^e^ (n)	Cost per result (Aus $)	Post reactions (n)	Post saves (n)	Post shares (n)
**Meta (Instagram and Facebook)**													
	February 10, 2023	February 28, 2023	18	Post engagement	14,128	36,340	112.96	10	68	0.03	3184	5	1
	March 2, 2023	March 8, 2023	6	Link clicks	88,929	138,404	59.87	10	550	0.11	28	4	17
	March 31, 2023	April 15, 2023	16	Link clicks	70,992	164,879	115.66	10	675	0.17	76	5	19
	April 28, 2023	May 12, 2023	15	Link clicks	86,464	237,153	143.3	10	775	0.18	106	6	22
	May 16, 2023	May 28, 2028	13	Link clicks	93,409	237,016	123.73	10	641	0.19	78	2	9
	May 31, 2023	June 14, 2023	15	Link clicks	117,056	345,260	144	10	680	0.21	208	1	9
	June 15, 2023	June 30, 2023	15	Link clicks	110,532	307,182	162.05	10	706	0.23	127	1	10
	July 4, 2023	July 18, 2023	14	Link clicks	96,816	301,837	143.73	10	664	0.22	69	3	8
	July 21, 2023	July 25, 2023	4	Link clicks	72,945	124,063	80.86	20	731	0.11	113	13	23
	July 28, 2023	July 31, 2023	4	Link clicks	64,672	99,327	59.77	20	410	0.15	66	7	8
	September 1, 2023	September 4, 2023	4	Link clicks	49,600	76,721	59.99	20	375	0.16	41	6	9
	October 13, 2023	October 16, 2023	4	Link clicks	44,795	72,066	59.99	20	435	0.14	51	7	14
	November 23, 2023	November 25, 2023	3	Link clicks	18,484	18,995	40	20	72	0.56	3	0	3
	December 1, 2023	December 3, 2023	3	Link clicks	35,278	36,989	41.18	20	85	0.48	0	0	0
	January 16, 2024	January 21, 2024	6	Link clicks	8185	13,463	62.74	15	103	0.61	3	0	3
	February 2, 2024	February 5, 2024	4	Link clicks	15,311	19,940	44.35	15	51	0.87	10	1	1
	February 22, 2024	February 23, 2024	2	Link clicks	114,365	134,196	24.45	20	190	0.13	61	1	0
	Totals	N/A^f^	146	N/A	408,077	2,363,831	1478.63	N/A	7211	N/A	4224	62	156
**TikTok**													
	August 25, 2023	August 29, 2023	4	N/A	8386	14,832	39.97	10	144	0.28	7	0	0
**Twitter**													
	February 14, 2024	N/A	N/A	N/A	N/A	1041	0	0	15	0	15	0	11

a Post engagement: strategy aims to encourage users to like, share, comment on, or save the advertisement. Link clicks: strategy aims to encourage users to click on the advertisement URL link.

b Number of times the advertisement is delivered to a unique account.

c Number of times the advertisement is delivered in total (including being delivered multiple times to one account).

d The conversion rate over the time of the study was approximately Aus $1=US $0.65.

e Number of times the advertisement URL link was clicked.

f N/A: not applicable.

### Progression of Participants From Screening to Enrollment

**Figure 1.**
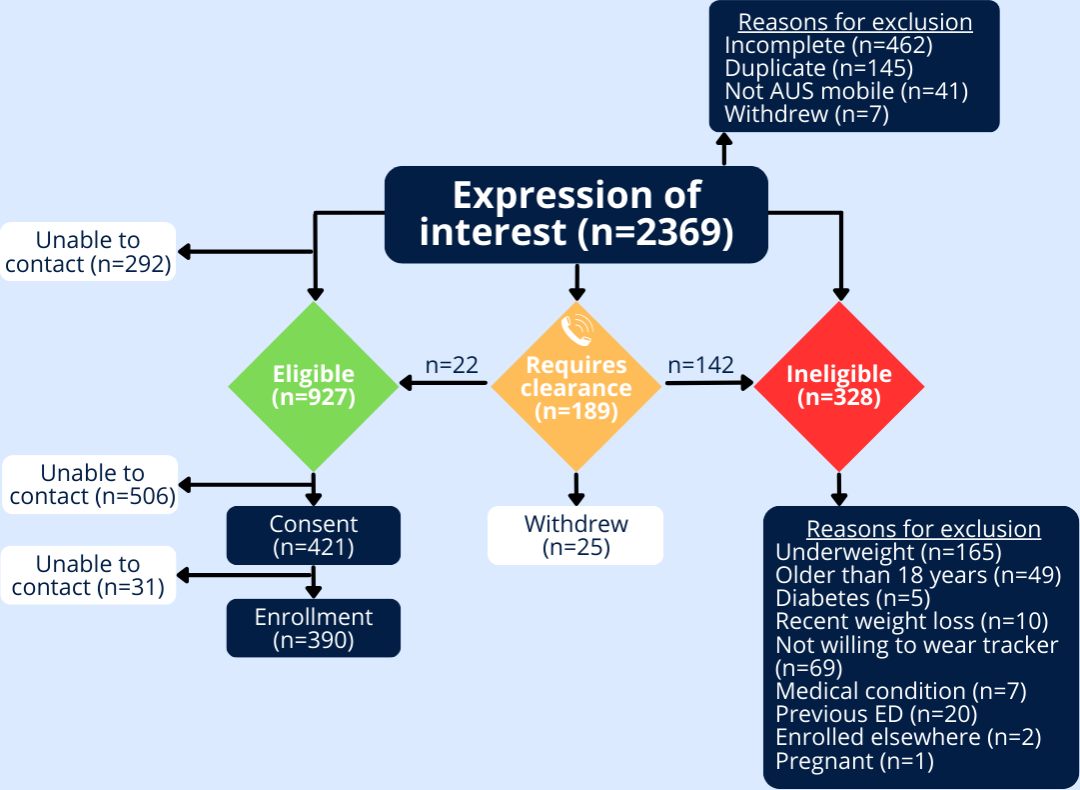
Progression of participants from screening to enrollment. AUS: Australian; ED: eating disorder.

Figure 1 shows the progression of participants and dropout points from screening to enrollment. Of the 2369 entries on the EOI form, 655 were immediately excluded. Of the excluded entries, 41 did not have an Australian mobile number. These 41 entries were among the first 100 EOIs and were believed to be bots. After adding a reCAPTCHA to the REDCap screening survey, no further suspicious entries were received. Of the remaining, 462 entries were incomplete, 145 were duplicate entries, and 32 withdrew their EOI postscreening. In addition, 328 entries were ineligible, as they did not meet the inclusion criteria, with reasons for exclusion shown in Figure 1.

A total of 481 potential participants scored above the cut point on the IOI-S. A total of 292 participants were unable to be contacted to complete the EDE-Q (per protocol for further screening for eating disorder risk). A further 189 potential participants were referred to the InsideOut Institute for assessment from eating disorder expert clinicians. Of those, 104 were unable to be contacted by the eating disorder expert clinicians (and were therefore marked as ineligible), 38 were ineligible postcall as they were assessed as high risk. A further 25 potential participants withdrew at this step, and 22 potential participants were assessed as eligible by the psychologist and sent the e-consent form.

A total of 927 potential participants were eligible and sent the e-consent form. Of those, 506 were unable to be contacted and the e-consent form was never signed. The e-consent form was signed by 421 participants, and they were sent the baseline surveys, but 31 participants did not commence answering baseline questions, giving 390/2369 (16.4%) as participants enrolled in this study.

### Factors Associated With Nonparticipation

During screening, 537 potential participants were identified as eligible but did not proceed to enrollment, and 390 participants enrolled in the Health4Me Study. Chi-square tests indicated that there were statistically significant differences in screening characteristics between eligible participants who did and did not enroll in this study for gender and recruitment method. Gender differences were significant (*χ*^2^_3927_=9.8, *P*=.02); ASRs indicated fewer males and more individuals reporting “other” enrolled than expected by chance alone. Additionally, the recruitment method was significant (*χ*^2^_3925_=17.39, *P*<.001), ASRs indicated fewer participants enrolled through Instagram and more enrolled through other methods (eg, known networks or word of mouth) than expected by chance alone. No differences were observed for other screening characteristics. A full breakdown of screening characteristics between those who were eligible and did or did not enroll is available in Table 3.

**Table 3.** Comparison of screening characteristics between those who were eligible who did and did not enroll in the Health4Me Study.

	Total eligible and not enrolled (n=537)	Total enrolled (n=390)	Chi-square (*df*)	*P* value
**Age (years)**			3.8 (1927)	.05
12‐14	80	41		
15‐18	457	349		
**Gender**			9.8 (3927)	.02^a^
Male	166	92		
Female	347	274		
Other	11	17		
Prefer not to say	13	7		
**BMI** ^**b**^			2.1 (3878)	.55
Underweight	21	16		
Healthy weight	410	279		
Above a healthy weight	60	51		
Well above a healthy weight	21	20		
**Attending high school**			0.5 (1927)	.82
Yes	464	339		
No	73	51		
**Recruitment strategy** ^**c**^			17.3 (3925)	<.001^a^
Facebook	18	22		
Instagram	470	301		
Other social media platform^d^	4	5		
Other^e^	44	61		

a Statistically significant.

b Due to being asked gender during screening and not sex assigned at birth, we are unable to accurately calculate BMI for those who listed their gender as “other” or “prefer not to say.”

c One record missing from each for recruitment strategy. Total eligible and not enrolled (n=536), and total enrolled (n=389).

d Due to small numbers, categories of Twitter/X, TikTok, and other social media platform were combined.

e Due to small numbers, categories of headspace, general practitioner or doctor, and other were combined.

## Discussion

### Principal Results

The Health4Me Study aimed at improving physical activity and nutrition behaviors among those aged 12 to 18 years. A total of 2369 EOIs were received, and 390/2369 (16.4%) participants were recruited in less than 12 months. Social media was the main source of recruitment. The research team did try to engage with schools, known networks, and relevant youth organizations via emails with limited success. Social media advertisements through Meta were effective, reaching 408,077 unique accounts across all states and territories in Australia. Overall, social media advertisements were low cost (Aus $3.89 per participant enrolled [approximately US $2.58]). From screening to enrollment, there were multiple points of dropout. Of the EOIs from potential participants who were eligible (927/2369, 39.1%), statistically significant differences were observed for those who did and did not enroll in terms of gender and recruitment method. Fewer males and more individuals reporting their gender as “other” enrolled than expected by chance alone. In addition, fewer individuals enrolled through Instagram and more enrolled through other methods (eg, known networks or word of mouth) than expected by chance alone.

### Comparison With Prior Work

Virtual clinical trials have the potential to address challenges in traditional site-based recruitment and be cost-effective [20]. Yet, prevention programs among adolescents are known to have the lowest recruitment rates [35], and stakeholders have identified that a prevention lens may not be engaging for adolescents [4]. In the Health4Me Study, a digital preventive intervention, digital recruitment strategies that were employed were effective, recruiting 390 adolescents in less than 12 months. The Health4Me Study was guided by factors associated with successful recruitment from a previous virtual clinical trial [36], including (1) national recruitment, (2) self-referrals, (3) unmet need for trial intervention, (4) patient and public involvement, (5) regular monitoring and communication, and (6) reimbursement and early exclusion. In the Health4Me Study, a national sample was recruited and participants self-referred into this study. In addition, there are limited prevention programs currently available for adolescents [37], and the intervention and all advertising materials were co-designed with adolescents [29]. A small day-to-day research team was employed who communicated regularly through detailed screening and recruitment logs, and participants were reimbursed through online gift vouchers after completing all study activities at baseline and 6-month follow-up.

The costs reported in this study for social media advertising are lower per enrolled participant than what has previously been reported in reviews (approximately US $3-US $628) [9,38], however these studies mostly report on Facebook and compare social media to traditional in-person recruitment. Limited research is available reporting virtual clinical trial recruitment costs to recruit adolescents. A virtual clinical trial, which aimed to prevent and reduce cyberbullying among adolescents that used Instagram for study recruitment, found a higher consent rate than the Health4Me Study (24.4% vs 16.4%) yet had much higher social media advertisement costs (approximately US $19 versus approximately US $2.59 per enrolled participant) [39]. It is essential for future virtual clinical trials to report costs associated with recruitment to understand their cost-effectiveness for enrolling participants from the target population.

Virtual clinical trials allow remote access to research, potentially enhancing the diversity of participants, and recruiting from hard-to-reach populations [20]. In the Health4Me Study, it was observed that more individuals reporting their gender as “other” enrolled than expected by chance alone. This “other” category captures any gender other than male or female (eg, nonbinary or transgender). In another virtual clinical trial targeting cyberbullying found that nearly half of the participants recruited via Instagram identified as lesbian, gay, or bisexual [39]. It was also observed that less eligible males enrolled in the Health4Me Study than expected by chance alone. When looking at the social media advertisements, they reached less males overall. Evidence from large datasets demonstrates that females spend more time overall and more time per day on social media [40,41]. Additionally, another virtual clinical trial among an older population found that males were underrepresented [42]. Future efforts should be directed to identifying effective methods to recruit males to virtual clinical trials.

Another factor considered to attract hard-to-reach participants online is that those interested can self-refer into this study. A previous study, which aimed to assess effectiveness of online behavioral therapy for tics among young people, found that the majority of participants self-referred from online [36], enabling those who were not under the care of mental health clinicians to be included. The Health4Me Study is unique in that participants who are aged 15 years and older can consent themselves into this study, without the need for parent or guardian consent. This was approved by the ethics committee, with support from our youth advisory group as the Health4Me Study is a low-risk, preventive health intervention. This capacity to self-refer gives adolescents some autonomy around their health, especially given that preventive care is seldom given within primary care to this age group [43].

Within this study, less eligible adolescents enrolled through Instagram than were expected by chance alone, and more enrolled through other methods (eg, known networks or word of mouth) than what was expected by chance alone. Hypothesized reasons for this are around trust in health information that adolescents view online, adolescents being discouraged from sharing personal information online, and having poor knowledge and attitudes on clinical trials [44]. Previous reviews show that adolescents often distrust health information found online yet continue to engage with this information [45]. In terms of health information on social media, friends and networks are particularly important for gaining adolescents trust in this space [46]. Future studies planning to recruit adolescents through social media could explore the use of peer referral or endorsement from youth advisors or reputable organizations (eg, study sponsor) to gain an increased level of trust. In addition, adolescents are acutely aware of how their personal data are being used, and building trust and authenticity among this population is vital [31]. Partnerships with known youth health organizations and endorsement of the clinical trial through their own social media accounts may be useful to increase trust among adolescents.

Within the Health4Me Study, there were multiple points of dropout from screening to enrollment. The largest point of dropout was those who were eligible and sent the consent form but never responded. For all eligible participants, the research team sent the consent form twice via email, however after no response they were marked “unable to contact.” Though emails are a highly acceptable form of communication among adolescents [47], future efforts should be directed to streamlining processes of screening and consent for scalability of future trials. Previous studies have aimed to do this using mobile apps, for example, ResearchKit (Apple Inc) [48], which is an open-source software framework designed to streamline the process of screening and consenting participants into research studies. Evidence of success is available for research studies among adults [49,50], yet no outcomes are currently available among adolescents [51]. Other strategies for enhancing communication with adolescents could also be explored in the future (with appropriate ethical approval), such as text messaging and direct messaging on social media platforms.

The second highest rate of dropout among participants was those who required further screening for a potential eating disorder. Out of 2369 potential participants, 292 (12.3%) did not complete an EDE-Q and were therefore excluded, and 189 (8.0%) required clearance through a phone call with this study’s psychologist. When compared to the prevalence of eating disorders overall among Australian adolescents, this rate is lower than what has previously been reported (point prevalence of 22.2%) [52]. Thus, screening for eating disorders was not identified as a barrier to enrollment, rather an important safety precaution for potential identification of disordered eating among this population in a preventive intervention.

### Limitations

Limitations in this study exist. First, this study is not representative of all adolescents due to inclusion criteria, which remove some groups. However, as this is a prevention intervention, the inclusion criteria aim to represent a large percentage of the adolescent population within Australia. Second, there are restrictions on advertising to adolescents via social media and changes are constantly occurring in this space. Though the inclusion criteria for age in this study was those aged 12 to 18 years, social media advertisements are unable to be targeted to adolescents aged younger than 13 years, as you can only establish a social media account if you are over 13 years. Advertisements were developed for distribution on Snapchat; however, advertising of clinical trials is not allowed on its platform. Therefore, recruitment of adolescents via social media is also a limitation to reaching adolescents aged <13 years. Third, adolescents report that they find recruitment via social media to be feasible and acceptable for recruitment and retention [53,54]. However, this was not assessed within this study as follow-ups are ongoing. Adolescent perceptions for using social media for recruitment will be assessed in the process evaluation for the Health4Me Study, by assessing retention rates and analyzing focus group data. The findings of this study require validation with studies among other adolescent populations and other types of interventions.

### Conclusions

Within the Health4Me Study, it was observed that recruitment was most effective via social media, and this was low cost per participant enrolled. Throughout the screening to enrollment process, there were multiple points of dropout, and future efforts should be directed toward streamlining screening and enrollment processes for scalability of future trials. In addition, our results highlight the importance of building trust among clinical trials and health information generally among adolescents on social media for future success in recruiting adolescents via this digital strategy.

## Supplementary material

10.2196/62919 Multimedia Appendix 1 Health4Me social media advertisement examples.

## Abbreviations

ANZCTR: Australia New Zealand Clinical Trials Registry
ASR: adjusted standardized residual
EDE-Q: Eating Disorder Examination Questionnaire
EOI: Expression of Interest
IOI-S: InsideOut Institute Screener
NSW: New South Wales
REDCap: Research Electronic Data Capture
