# Supplementary figures and images for: Recruitment of Adolescents to Virtual Clinical Trials: Recruitment Results From the Health4Me Randomized Controlled Trial
Source: JMIR Pediatr Parent. 2024 Dec 16;7:e62919. doi: 10.2196/62919 (PMC11683508; doi:10.2196/62919)

#### **
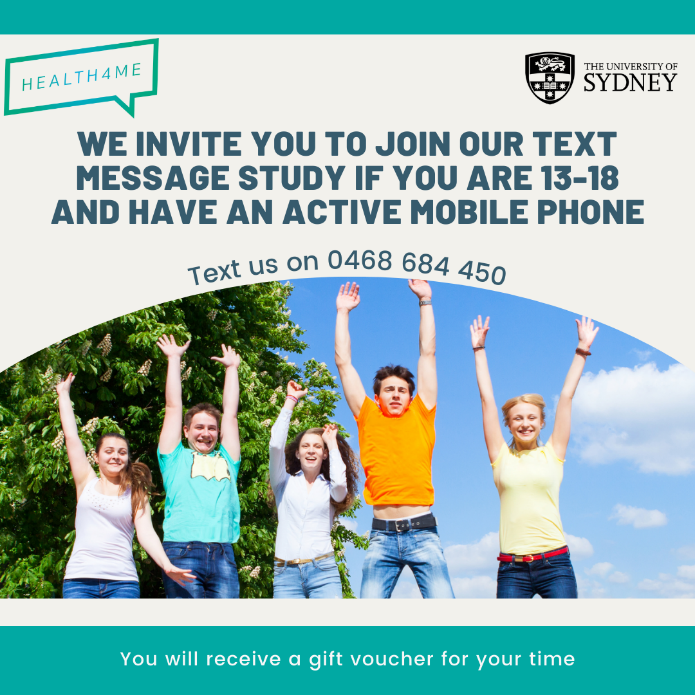

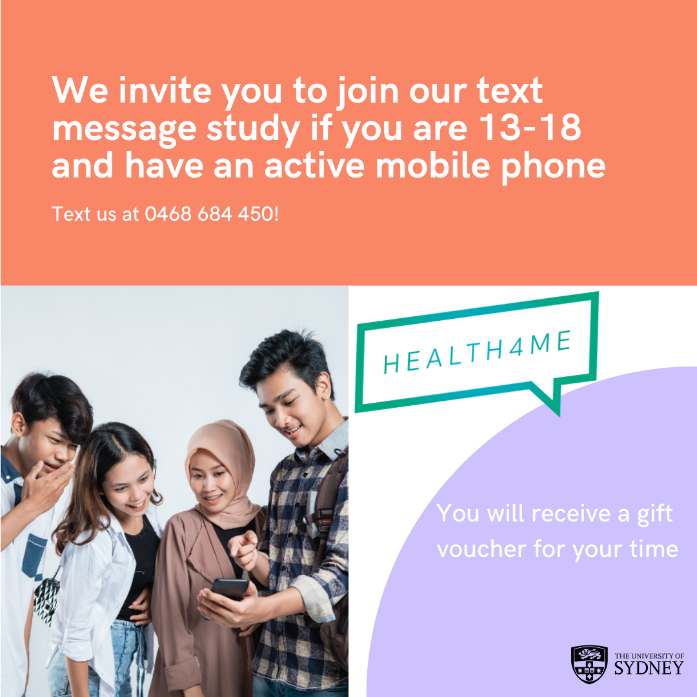

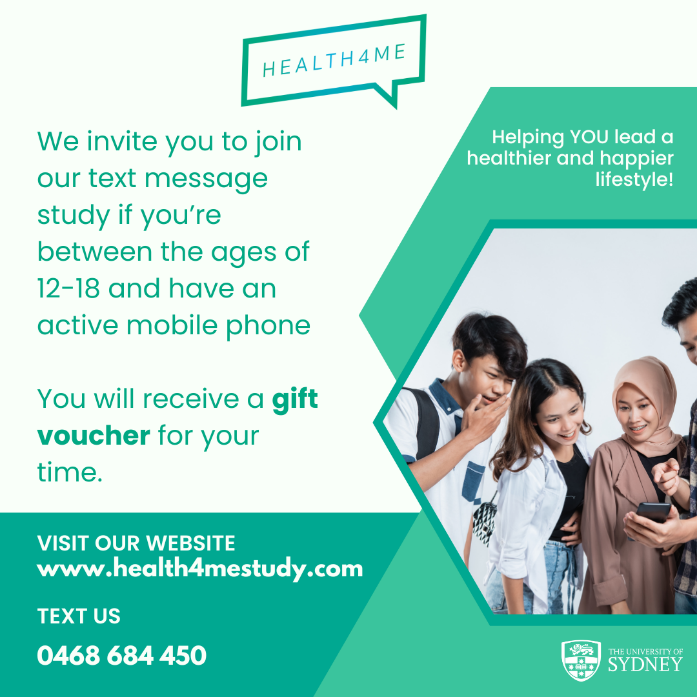

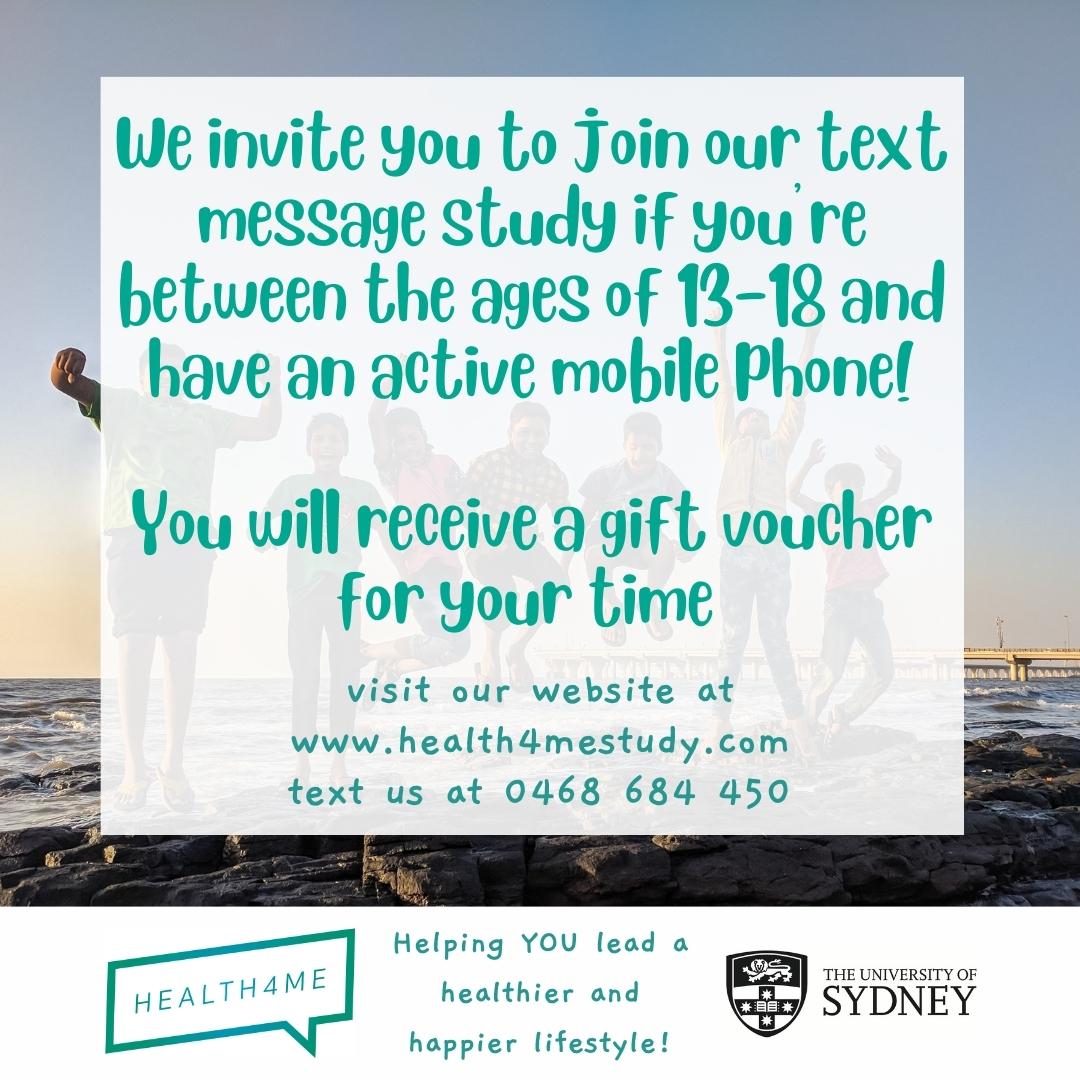
**

Supplement: Multimedia Appendix 1 [file pediatrics-v7-e62919-s001.docx]
